# Perceptions of Dutch general practitioners towards eHealth for patients with type-2 diabetes: a qualitative study

**DOI:** 10.1093/fampra/cmac066

**Published:** 2022-06-25

**Authors:** Lieve Vonken, Hani Hussein, Rik Crutzen, Stan Vluggen

**Affiliations:** Maastricht University, Care and Public Health Research Institute, Department of Health Promotion, PO Box 616, 6200 MD Maastricht, The Netherlands; Maastricht University, Care and Public Health Research Institute, Department of Health Services Research, PO Box 616, 6200 Maastricht, The Netherlands; Maastricht University, Care and Public Health Research Institute, Department of Health Promotion, PO Box 616, 6200 MD Maastricht, The Netherlands; Maastricht University, Care and Public Health Research Institute, Department of Health Services Research, PO Box 616, 6200 Maastricht, The Netherlands

**Keywords:** adherence, adoption, diabetes, eHealth, general practitioners, innovation

## Abstract

**Background:**

eHealth provides a viable option to facilitate type-2 diabetes mellitus self-management and adherence. To this end, a web-based computer-tailored eHealth programme, My Diabetes Profile (MDP), was developed and implemented in Dutch diabetes care. To fully utilize the potential of eHealth, the reach of effective programmes like MDP should be maximized. Therefore, it is vital to explore perceptions of general practitioners (GPs) regarding eHealth and factors that influence GPs’ decision to adopt eHealth programmes.

**Objective:**

To shed light on Dutch GPs’ perceptions towards eHealth in general and specifically, the adoption of MDP.

**Methods:**

Interviews were conducted among a heterogeneous sample of 16 Dutch GPs. The interview guide, based on the Diffusion of Innovations Theory, addressed perceptions about eHealth in general, characteristics of MDP, organizational characteristics, and external influences on adoption. Audio-recordings were transcribed and analysed using deductive coding in NVivo.

**Results:**

Nearly all GPs used some form of eHealth and listed many benefits and few drawbacks about eHealth. Sometimes, GPs were unaware of what eHealth encompassed; programmes resembling MDP were not mentioned. COVID-19 immensely increased eHealth uptake, especially for remote communication. Regarding MDP, the organizational and external influences on adoption were limited, while characteristics of the innovation were deemed more important. GPs expressed benefits of MDP (e.g. uncomplex, user-friendly, tailored) other than attributed to eHealth in general and fewer drawbacks.

**Conclusion:**

While GPs’ opinions about eHealth and MDP were positive, the concept of MDP was relatively unfamiliar. Future research should focus on targeting GPs’ awareness of eHealth possibilities.

**Key messages**

- Nearly all GPs use eHealth and they see many benefits of eHealth.
- Sometimes, it was unclear to GPs what eHealth encompassed.
- COVID-19 was perceived to have immensely increased eHealth uptake.
- Innovation characteristics (e.g. effectiveness) seem to influence adoption.
- The organizational and external influences on eHealth use seem limited.

## Introduction

Worldwide, over 500 million people live with type-2 diabetes mellitus (T2DM), and it is anticipated that almost 800 million people will be living with it by 2045.^1^ In the Netherlands alone, T2DM, affects approximately 1 in 17 people.^2^ T2DM treatment focusses on lifestyle adaptations such as improving diet and activity levels. However, ultimately, patients may require medical interventions such as oral glucose-lowering drugs or insulin injections.^2^ The majority of patients do not meet these lifestyle recommendations, and prevalence of medication adherence is often below 80%.^3^ Moreover, nonadherence within treatment elements often coincides, thereby attenuating the potential of treatment effects.^4^

In Dutch primary care, the main and supervising healthcare providers for patients with T2DM are general practitioners (GPs) who assist patients in treatment strategy formation and adherence. There are also brief quarterly check-ups with practice nurses who specialize in chronic care.^5^ Whether the number, duration, and generic nature of these check-ups is enough to prompt behavioural change, treatment adherence, and self-management is debatable.^3^ Specifically, in the Netherlands, the application of successful methods to increase treatment adherence, such as goal setting, patient preparation, and shared-decision making, has been described as suboptimal.^6^ Hence, successful adherence largely depends on patients’ continued self-management, which is hindered by the dynamic and chronic nature of T2DM (i.e. patients must constantly adapt to the multiple factors influencing glucose levels).^7–9^

In order to facilitate diabetes self-management, eHealth provides a viable option by providing continuous and tailored support to optimize patients’ adherence.^10^ eHealth programmes have already been used successfully in patients with T2DM to improve glycemic control and treatment adherence,^4,11–13^ increase health-related quality of life, and decrease healthcare costs.^14^ In recent years, a web-based computer-tailored eHealth programme—My Diabetes Profile (MDP)—has been developed and implemented in Dutch T2DM care. By offering self-management support, MDP aims to improve patients’ adherence to lifestyle and medical recommendations.^15^ What is unique about MDP are the insights provided into personal risk behaviours and the continued and iterative feedback on changing a chosen behaviour, tailored to relevant determinants. Moreover, MDP was developed in a co-creative fashion, applies goal-setting principles and tracking tools, and is mainly visually oriented.^15^ After their GP or practice nurse registers them in the programme, patients can work in it independently. The GP or practice nurse can then review their patient’s activity in the programme and discuss it with them in subsequent consultations.

In a nationwide cluster randomized controlled trial, MDP was effective in improving overall treatment adherence in patients with T2DM, reflected by a small-to-medium effect size.^4^ To fully utilize the potential of eHealth for T2DM, the reach of effective eHealth programmes like MDP should be maximized by ensuring wide-scale adoption and implementation.^16^ However, the uptake of eHealth for self-management purposes remains limited in Dutch medical care.^17–19^ Therefore, it is vital to explore and understand GPs’ perceptions towards eHealth and factors influencing their decision to adopt self-management eHealth programmes for their patient population.

The Diffusion of Innovations Theory by Rogers is among the most established theories used to understand factors influencing the implementation of innovations. This theory highlights the importance of exploring salient characteristics of innovation, organization, and external influences and the innovativeness of the adopter as determinants for adoption of interventions.^20^ Several reviews provide a valuable generic overview of relevant barriers and facilitators to the adoption and implementation of a wide array of eHealth programmes amongst healthcare providers.^21–24^ The barriers and facilitators identified in these reviews are similar to the categories of the Diffusion of Innovation Theory and include, for example, stakeholder acceptance,^23^ and integration and interoperability.^22^ However, the innovation-decision process is highly dependent on the specific nature of a certain eHealth programme, as well as the adopter’s awareness, knowledge and attitude of an innovation.^20^ Therefore, it is vital to study GPs’ perceptions specifically regarding MDP adoption.

The research question of this study is: What are perceptions of Dutch GPs towards eHealth for patients with Type-II Diabetes and, in particular, about MDP? These insights can be used in tailored strategies to enhance adoption, reach and impact of proven effective eHealth programmes.

## Methods

### Study design

This study employed a qualitative design using semistructured individual interviews with Dutch GPs. The study was approved by the Maastricht University Faculty of Health, Medicine and Life Sciences Research Ethics Committee (FHML/HPIM/2020.039) and follows the Consolidated Criteria for Reporting Qualitative Research (COREQ).^25^

### Recruitment and procedure

GPs were recruited from April to June 2020 through an overview of national GP practices, networks of the researchers involved in the study (HH and SV), LinkedIn, Facebook, and snowball sampling. To extend the degree to which results can be generalized and to reflect a wide array of perspectives, efforts were made to recruit a heterogeneous sample in terms of age, gender, working experience, geographical area (urban vs. rural), and practice type (community vs. separate). No initial target number of GPs was set, however, after 14 interviews no new interview themes could be generated. Two additional interviews were carried out to confirm content saturation and validity.^26^ The eligibility criteria for GPs were: (i) being a GP (or in the final phase of medical training) employed in the Netherlands and (ii) speaking English. GPs who participated in the nationwide effectiveness trial were excluded.

After initial contact, GPs received study information and participatory conditions by email and could consider participation for 1 week. After consent via email, an interview was scheduled. Interviews were pilot tested to last approximately 30 min. Due to COVID-19-related contact restrictions, interviews were conducted via telephone or Zoom. Before the interview, GPs received a 1-page information document, a visual representation of MDP, and a brief questionnaire to identify personal characteristics. The questionnaire contained questions on the participant’s age, gender (male/female/other), years of being employed as a GP, practice environment (rural/urban), practice type (community/separate) and size, and association with a regional GP network (yes/no). Participants were unfamiliar to the researchers conducting the interview (HH: female, MSc, English speaking research intern; or SV: male, PhD, senior researcher) and before interview commencement, briefly, time was taken to get acquainted. Subsequently, interviewees were requested to verbally reinforce consent for participation and audio-recording of the interview, after which the semistructured interview was conducted, guided by an interview guide (Supplementary file 1). Participants were informed that the transcript would be available on request to add potential comments.

### Measurements and theoretical concepts

The interview guide was based on the Diffusion of Innovations Theory.^20^ The theory highlights the importance of exploring adopter characteristics, characteristics of the innovation itself, organizational characteristics in which the innovation is to be implemented, and external influences. To gain insight into the adopter characteristics, interviewees were asked about their current eHealth use and personal and normative perceptions about eHealth in general. Second, GPs’ perceived characteristics of MDP, the innovation, were mapped. This included the attributes relative advantage, compatibility, complexity, trialability, and observability. Third, organizational characteristics which may influence MDP adoption were explored. For example, the innovation-system fit or the influence of other decision-makers within an individual’s organization. Last, the potential external influences on MDP adoption were explored. This included questions on regulations or reimbursements related to the adoption of eHealth on a national or regional level.

### Analysis

Interview recordings were transcribed verbatim and coded deductively using NVivo 12 software.^26^ A predefined coding tree was applied (Supplementary file 2), based on the constructs and subconstructs of the Diffusion of Innovations Theory.^20^ The main constructs were related to the characteristics of the adopter, innovation, organization, and external influences. In turn, subconstructs were put under the umbrella of these main constructs (e.g. adopter characteristics were subdivided into current eHealth use, perceptions on eHealth, and normative beliefs; innovation characteristics were subdivided into the subconstructs relative advantage, complexity, compatibility, trialability, and observability). First, interview transcripts were read closely, and text segments were coded by HH under constructs or subconstructs of the Diffusion of Innovations Theory. After completion, SV and LV reviewed the coding of all interview transcripts and revised the coding if deemed appropriate, in order to validate the initial coding of HH. Only a few minor adjustments were made by authors SV and LV, for example, some text segments were coded under a different (sub)construct after discussion with the research team. No new codes were added to the initial coding tree. Reoccurring themes throughout the codes were described following the (sub)constructs from the Diffusion of Innovations Theory. Quotes were added, specifying the participant number.

## Results

Sixteen Dutch GPs from across the Netherlands were interviewed, with an average age of 41 and an average working experience of 16 years, see Table 1. The geographical area of the practice and practice type was rather evenly distributed. On average, interviews lasted 38 min. No participant requested the transcribed interview to add potential comments.

**Table 1.** Sample characteristics of interviewees (2020): 16 Dutch general practitioners.

	*N* (%)	Mean (SD)
GP characteristics		
Gender		
Male	7 (43.8)	
Female	9 (56.2)	
Geographical area of practice		
Urban	8 (50)	
Rural	6 (37.5)	
Practice type		
Community practice	5 (31.3)	
Separate practice	7 (43.8)	
Practice part of regional GP network		
Yes	12 (75)	
No	2 (12.5)	
Age (years)		41 (11.1)
Work experience as a GP (years)		16 (9.4)
Number of registered patients in practice		3,563 (1,893.8)

### Adopter characteristics and current eHealth use

#### Current eHealth use

Almost all GPs mentioned using some form of eHealth, without being provided with a definition or common examples. GPs indicated to use eHealth for a variety of purposes. They applied it in phone, video, and email consultations, for reviewing patient data, and for making appointments. eHealth was most often applied in the fields of dermatology and mental health, and for monitoring of glucose levels. However, it seems that GPs often underestimated their eHealth usage due to uncertainty about what eHealth comprised.

I don’t know if you can call it eHealth, but our patients are able to communicate and ask us questions about their health by e-consults. […] We also work a bit with pictures within dermatological diseases. We are now starting with e-consults or video chats for diabetes patients who also have the opportunity to note glucose measurement and they are monitored online. [P4]

Half of the GPs indicated that the COVID-19 pandemic immensely increased the uptake of eHealth. For almost every GP, the pandemic made the shift from traditional patient–doctor consultation to e-consults inevitable. For some GPs, this led to the revelation that face-to-face counselling was not always required.

The great discovery of this Corona crisis is that for a large number of cases physical contact with a doctor isn’t necessary. […] In chronic illnesses it’s almost totally unnecessary. [P2]

#### Perceptions on eHealth in general

GPs put forward many advantageous beliefs concerning eHealth in general. Most frequently, eHealth was considered efficient, in terms of workload, ease of use, costs, and time, and flexible, given its constant availability.

I think it’s efficient for your practice, efficient for the patients, you no longer need to wait in the waiting room, you can see your own figures, I think it’s very good for patients to know their own figures and see their charts. But I think it’s necessary to also have personal contact. [P8]

In addition, eHealth was believed to facilitate disease management, patients’ autonomy, and the monitoring of patients at home. Less often it was mentioned that eHealth could improve communication and the doctor–patient relationship and could contribute to a solution for the lack of physicians. The drawback of eHealth heard from most GPs entailed that eHealth was not considered suitable for everyone, for example, excluding people characterized as older, less (health) literate, and those who do not use the Internet.

There’s a big part of the population that doesn’t tend to hook up with these kinds of projects. It’s especially these kinds of group you want to reach because they experience the most problems. […] It’s very good, but I think we need to [ensure] that eHealth isn’t something that divides society. [P3]

Generally, eHealth was viewed as a tool rather than a solution. Almost all GPs believed that personal contact was still required, as eHealth was sometimes considered impersonal and as a form of contact only providing a partial impression.

Because of the Corona crisis we do a lot more consultations by email. They [patients] email us questions with for example a picture of their skin. […] That’s very new for me and I think it can be helpful but only if it’s impossible to do a physical consultation. I don’t think eHealth will ever be a complete substitute for seeing a doctor. [P6]

Some GPs indicated that while eHealth would save time in the long run, it would cost them time at first. Other infrequently mentioned disadvantages were the overkill of available apps and patients not using eHealth as intended (e.g. lying about their self-monitored data).

Of course it takes time. You have to get training in it. You have to train older people in the clinic with it, so it’s something I would think about because we have little time, and you need to learn new stuff so maybe that’s a disadvantage. [P5]

#### Subjective norms

When asked about whether colleagues of the interviewed GPs used any form of eHealth, GPs indicated mixed use. Although no GP mentioned pressure from colleagues to use eHealth, some indicated that colleagues, supervisors, or education inspired them to use specific programmes. Generally, COVID-19 and related contact restrictions made eHealth use more “normal” for GPs.

We had those pilots and then there were, I think, 50 doctors, and 40 of them were really interested and wanted to start with it. And then I thought, oh, quite interesting. I’ll wait a little bit for the experiences of the others. [P10]

One GP, however, advocated for a general mindset change to enable eHealth implementation.

The biggest problem is of course [a] mindset change, especially on the healthcare workers’ [side] and not on the patients’. Because patients are actually used to all kinds of electronical solutions in their normal life. […] The problem is on the side of the healthcare workers. [P2]

### Innovation characteristics: perceptions on MDP

In general, GPs indicated to be relatively willing to adopt MDP, reflected by an average score of 7.4 on a scale from 1 to 10.

I think it’s very interesting because when I read it, I was like: “Oh yeah, of course it’s going to be diabetes again!” […] But then I saw it was more about the lifestyle. I thought that was very interesting, and I think it’s very practical and I like it. I said in the beginning; I struggle with clear examples for what eHealth is and what you can do with it, and this was for me, a new way to think about it and what you can do with it. [P6]

#### Relative advantage

When asked specifically about MDP, GPs’ perceived advantages differed from those mentioned for eHealth in general. The most frequently mentioned advantageous attributes were its visual orientation, ease of use, the ability to provide patients with tailored care, and the possibility for patients to select a focus behaviour autonomously. The constant availability and possibility to monitor patients at home were also viewed as benefits.

I think it’s good to focus on one thing and not too many other things and [that] people have a choice as to where they want to focus on. [P3]

Several GPs viewed MDP as a tool to involve patients in their disease management. Some GPs believed that the insights patients got into their values and the frequent feedback the programme offered—compared with regular consultations—led to improved health outcomes. A few GPs indicated to consider using MDP if it would be better than current practice. Only a few GPs mentioned potential drawbacks or concerns, such as MDP’s compatibility with other eHealth programmes and internal ICT systems, or what would happen if patients were not motivated to use the programme once the excitement had vanished.

What will happen when the patient doesn’t go to the computer and [doesn’t] start and log in. Will they get a reminder from the nurse or the doctor? [P10]

#### Complexity

All but 1 GP indicated that patient enrolment to MDP would be delegated to practice nurses as they were the primary contact persons for diabetes care and were believed to know the patients best. Implementation time issues, due to general time pressure, were mentioned by many GPs even though most viewed the programme as easy to use and navigate. About 1 in 3 GPs indicated possible issues related to the motivation and capabilities of practice nurses (e.g. not recognizing MDP’s added value, lacking familiarity with eHealth).

New things are always difficult in the beginning, in practices and also for patients. There will be groups that will make it easier and there will be groups who think “Yeah whatever, I will not use it, I’ll do whatever I want.” So, it depends on the patients, on age, and also on my practice nurse. [P1]

#### Compatibility

Almost all GPs put forward patient characteristics that reduced the compatibility of MDP. These included limited Internet skills or connection, lack of willingness for behaviour change, older age, or speaking a foreign language. To enhance compatibility with the target audience, more than half of the GPs believed that the patient should be involved in the decision to enrol in MDP. For example, user experience was deemed important; sometimes even more important than effectiveness. Several GPs advocated MDP should be integrated into existing (ICT) systems or other eHealth programmes, to not choke in the multitude of apps and available information. Moreover, 1 in 3 GPs believed that MDP should be applied in combination with regular care.

The most important part is the user experience; it has to be something people not only like but love to use. What you see now is that with eHealth solutions, the possibilities technology gives us make them very often very complicated. Because people are putting [in] quite a lot of questionnaires and whatever. […] And if it’s easy to use, it will be used. [P2]

#### Trialability

Almost all GPs preferred a demo version of MDP in considering adoption. However, several GPs mentioned specifically that this was not necessary for their adoption decision. GPs perceived MDP as low risk, user-friendly, having no adverse effects on their patients, and easy to discontinue. One GP stated to be interested in the feedback of patients during an experimental period.

#### Observability

Almost all GPs indicated that the effectiveness of MDP would influence their adoption decision. While the effectiveness of MDP had already been established, some GPs added that they wanted to judge the convincingness of the results with more information on the research design and long-term effects. A few GPs added that the effectiveness of MDP was self-evident to them and would thus be easy to explain to their patients.

I believed in it even before you mentioned the trial, so it doesn’t influence my answer. […] This is an app that I believe stimulates behavior and talks about behavior. […] This is information-based, so I guess there’s not a real trial necessary. [P15]

### Organizational factors

When asked explicitly about organizational factors influencing adoption, GPs mainly described who would make the adoption decision. The choice was often made by the GP, sometimes in collaboration with colleague GPs or the GP practice owner. In some cases, practice nurses or regional care authorities would be involved in the decision. Near half of the GPs considered a positive attitude of the practice nurse towards MDP as a prerequisite for adoption.

### External influences

Almost all GPs mentioned data protection regulation as an external barrier for adoption, highlighting the difficulty of obliging to all legal aspects. Moreover, near all GPs indicated that support from larger organizations (i.e. regional care authorities, GP networks, the Dutch Diabetes Fund) or funders (i.e. insurance companies, integrated care) was a facilitator or even a precondition for adoption. A few GPs mentioned that lack of reimbursement was not a barrier to adoption. One GP mentioned that the coverage of MDP by health insurers would be an important aspect for patients. Almost half of the GPs indicated experiencing no or little influence from their GP networks. Some GPs indicated not being aware of any external influences.

Sometimes you get some sort of financial compensation for the extra work you do if you test the application, or if you’re in a pilot group, but I haven’t seen any. […] I think for most doctors the financial part is not that important. As long as it doesn’t cost you extra time. […] If that’s not the case, it might solve less physical contact with the patient, while you still get the same amount of money for your chronic care. [P11]

The main results are summarized in Table 2.

**Table 2.** A summary of the interview results.

Adopter characteristics	Innovation characteristics	Organizational characteristics	External influences
*Current eHealth use* • High eHealth usage. • COVID-19 increased eHealth use. • GPs might underestimate their eHealth use. *Perceptions on eHealth in general* • Many advantageous beliefs. Few drawbacks. • eHealth is a tool and cannot replace face-to-face consultations. *Subjective norms* • Limited, sometimes new ideas were offered.	• Willingness to adopt. • *Relative advantage*: Different advantages than for eHealth in general. Few drawbacks. • *Complexity*: Implementation issues were mentioned though in most cases, practice nurses would do the implementation. • *Compatibility*: Is reduced by patient factors. MDP should be integrated in current (ICT) systems. • *Trialability*: Is high, demo sometimes requested. • *Observability*: Effectiveness influences adoption decision.	• MDP adoption often decided on by GP. • Attitude of the practice nurse is influential.	• Barrier: Data protection regulation. • Facilitator: Support from larger organizations or funders.

## Discussion

This study sheds light on Dutch GPs’ current eHealth use and their perceptions about eHealth in general and adopting MDP specifically. Nearly all GPs indicated using some form of eHealth and, in general, many benefits and few drawbacks were put forward. Sometimes, it was unclear to GPs what eHealth encompassed and programmes resembling MDP were not mentioned. COVID-19 was perceived to have immensely increased eHealth uptake. The social influence on general eHealth use seems limited to receiving inspiration from other GPs. In terms of MDP, the organizational and external influences on adoption also seem limited, while innovation characteristics were described to influence adoption. When compared with general eHealth, the benefits of MDP differed and fewer drawbacks were listed. MDP was considered easy to use and navigate, user-friendly, and low risk. Almost all GPs preferred a demo version when considering adoption. The effectiveness of MDP was deemed important; however, user experience and patient involvement were mentioned equally often. Last, compatibility issues were put forward (e.g. fit to the patient population, existing ICT systems, and implementation of MDP by practice nurses).

Our results suggest Dutch GPs use and are positive about eHealth, which is in agreement with the results of recent similar work conducted among Dutch GPs.^27,28^ The COVID-19 pandemic, which is recognized as an inevitable driving force of remote communication in previous research, has accelerated the use of eHealth.^29^ Indeed, teleconsultation with GPs has been acknowledged as time-efficient and not inferior to face-to-face consultation in studies focussed on GP–patient communication quality.^30,31^ It is also evaluated well by patients, especially for chronic diseases, where it facilitates self-management.^30,31^ The appropriate management of chronic diseases such as T2DM is facilitated by periodic interaction with healthcare providers. Although it can be debated whether GPs’ eHealth use was intrinsically motivated or bolstered by external events such as the pandemic, a lesson learned by necessity is that the ease of teleconsultations benefits both GPs and patients. Corbett et al. also describe that the COVID-19 pandemic elucidated the benefits of teleconsultations, specifically for chronic diseases.^32^

Teleconsultations are a good example of what GPs believe eHealth entails. While GPs’ general attitude towards eHealth was positive, their notion of it was often limited to process-facilitating programmes supposed to be used by the GPs themselves. Recent work also demonstrates that GPs more frequently use eHealth themselves, and less frequently facilitate its use among their patients.^33^ The question arises, therefore, as to whether GPs are aware of the broad spectrum of eHealth applications and possibilities they could use with and for their patients. To maximize the impact of effective eHealth interventions, their reach and adoption should be optimized.^34^ On the one hand, based on the current results, it could be argued that GPs’ knowledge about MDP, which has been recognized as an essential precursor for adoption decisions, is insufficient.^20^ On the other hand, once GPs were made aware of the existence of eHealth self-management support programmes such as MDP, they acknowledged its unique features. Moreover, GPs identified few drawbacks and expressed a willingness to adopt the programme. Bridging the persuasion phase, using MDP’s unique attributes, might thus be feasible.

Acknowledging positive attributes and indicating a willingness to adopt an innovation are important psychological predictors of adoption.^20,35^ However, these factors do not ensure efficient implementation and maintained use, because intention does not guarantee behaviour and positive outcome expectations might not be the only factor influencing willingness.^36,37^ The healthcare context has often been described as complex^38^ and organizational factors have been associated with eHealth adoption and implementation.^17^ For MDP, GPs admitted to believe that eHealth did not suit all their patients (e.g. because of language or literacy barriers). While GPs generally decide on MDP adoption independently, the support of the practice nurse was seen as a prerequisite for adoption. While perceived social norms have been related to the acceptance of eHealth programmes in inpatient care,^35^ this was not the case here. Moreover, few external or organizational barriers to adopting MDP exist in the Netherlands, though the impact of the new data protection regulation on healthcare organizations has been identified.^39^

### Limitations

In this study, the collected data gives a detailed insight into GPs’ opinions about MDP. However, in Dutch healthcare, GPs are not solely responsible for the adoption and implementation of a programme such as MDP. GPs, with responsibility for many different patients, might have a different attitude towards MDP than, for instance, a diabetes nurse in secondary care. These stakeholders should be involved in further research. To fully understand the influences of stakeholders on the adoption decision, the perspectives of practice nurses and patients should also be investigated. Lastly, a limitation to the generalizability of the results is that to participate, GPs had to have a specific command of the English language. This may have caused selection bias based on GPs’ perception of their own level of English.

Strengths of this study include the theoretical base that informed all stages of the research process (i.e. interview guide, coding tree, and reporting). The semistructured interviews facilitated comparison between the heterogeneous sample of GPs and to other studies. While this partly limited the potential results to those fitting this theoretical base, the in-depth qualitative interviews provided rich data above and beyond the constructs from this theory.

## Conclusion

Dutch GPs seem positive about eHealth programmes. Process-facilitating solutions (e.g. teleconsultations) were used often. Programmes like MDP, designed for assistance in self-management for chronic care patients, seem unfamiliar to GPs. Organizational and external factors seem to neither hinder nor facilitate eHealth adoption. As the GPs in the sample currently do not see the full potential of eHealth interventions, increasing awareness about eHealth possibilities is a possible target for eHealth adoption initiatives to ultimately increase the potential impact of such interventions for patients.

## Supplementary Material

cmac066_suppl_Supplementary_File_1

cmac066_suppl_Supplementary_File_2

## Data Availability

The data underlying this article cannot be shared publicly due to the privacy of individuals that participated in the study. The de-identified data will be shared at reasonable request to the corresponding author.
