# Supplementary material for: Perceptions of Dutch general practitioners towards eHealth for patients with type-2 diabetes: a qualitative study
Source: Fam Pract. 2022 Jun 25;40(1):91–7. doi: 10.1093/fampra/cmac066 (PMC9384395; doi:10.1093/fampra/cmac066)
Supplement: cmac066_suppl_Supplementary_File_1 [file cmac066_suppl_supplementary_file_1.docx]

**Supplementary file 1**

Interview guide: operationalization of constructs Diffusion of Innovation Theory

| Construct | Operationalization |
| --- | --- |
| Adopter characteristics | What is your general opinion on eHealth and using eHealth programs in healthcare?  Do you see any the benefits of using eHealth programs?  What, according to you, are the limitations of using eHealth?  To what extent do GPs in your network use eHealth initiatives?  To what extent do other GPs think you should use eHealth initiatives? |
| Innovation characteristics | Based on the program description, what are your initial thoughts and impressions of MDP?  What features of MDP do you like and not like?  Do you think it will be easy or difficult to implement MDP at your practice and why? What situations or circumstances would make it difficult?  Compared to the care currently given to diabetes patients, do you think MDP has any added value?  To what extent do you think MDP could easily be integrated in the current systems in place at your practice, i.e., how compatible is it with the standard practices?  What is the most important factor for you, when considering adopting a new eHealth initiative?  To what extent is a trial period an important factor when considering adopting MDP?  MDP has proven to be effective in improving treatment adherence in patients with T2DM in a large trial across the Netherlands. How does knowing this influence your idea of adopting it?  On a scale from 1-10, how likely would it be that you would adopt the MDP-program and why? |
| Organizational characteristics | Does your organization currently use any eHealth in the treatment of T2DM?  In your view, what could hinder eHealth use in your practice?  In your view, what could facilitate eHealth use in your practice?  Who are the key individuals in your practice or organization, who would make the decision to adopt MDP or other eHealth initiatives?  Would the adoption of these initiatives have to be discussed with others?  Would you rather recruit patients yourself or delegate this to a practice nurse, and why? |
| External influences | Are there any specific policies or regulations in the Netherlands around “the adoption of e-health initiatives” that facilitate or hinder adoption in your practice?  Within the GPs regional networks, are their social or formal rules that affect the adoption of eHealth technology?  Are there any reimbursement schemes and/or financial incentives for using eHealth systems? For example, health insurance reimbursement? |
