# Supplementary material for: Perceptions of Dutch general practitioners towards eHealth for patients with type-2 diabetes: a qualitative study
Source: Fam Pract. 2022 Jun 25;40(1):91–7. doi: 10.1093/fampra/cmac066 (PMC9384395; doi:10.1093/fampra/cmac066)
Supplement: cmac066_suppl_Supplementary_File_2 [file cmac066_suppl_supplementary_file_2.docx]

**Supplementary file 2**

Coding tree

1. **Adopter characteristics**
   1. Current eHealth use (e.g., intention to use eHealth and trends in eHealth adoption)
      1. Innovative
      2. Not innovative
   2. Perceptions of eHealth in general (e.g., attitudes, self-efficacy, awareness)
      1. Pros
      2. Cons
   3. Subjective norms (e.g., collegial use and their perceptions)
2. **Innovation characteristics**
   1. Overall adoption willingness
   2. Relative advantage
   3. Complexity
   4. Compatibility
   5. Trialability
   6. Observability
3. **Organizational characteristics**
   1. Organisational barriers to adoption
      1. Practice characteristics (i.e., workload, IT)
      2. Patient groups
      3. Medical professionals
   2. Organisational facilitators for adoption
      1. Training and support from the developer
      2. Practice characteristics (i.e., workload, IT)
   3. Solo vs group practice (regional networks e.g. ZORG Group)
4. **External influences**
   1. Government/laws/regulations
   2. Incentives/support
   3. Insurance companies (coverage/reimbursement)
   4. GP regional networks
